# Multimodal MRI for Estimating Her-2 Gene Expression in Endometrial Cancer

**DOI:** 10.3390/bioengineering10121399

**Published:** 2023-12-06

**Authors:** Xiwei Li, Shifeng Tian, Changjun Ma, Lihua Chen, Jingwen Qin, Nan Wang, Liangjie Lin, Ailian Liu

**Affiliations:** 1 Department of Radiology, The First Affiliated Hospital of Dalian Medical University, Dalian 116011, China; lxiwei2022@163.com (X.L.);; 2 Department of Pathology, The First Affiliated Hospital of Dalian Medical University, Dalian 116011, China; 3 Clinical and Technical Support, Philips Healthcare, Beijing 100016, China

**Keywords:** endometrial cancer, magnetic resonance imaging, amide proton transfer-weighted imaging, diffusion kurtosis imaging, T2 mapping

## Abstract

Purpose: To assess the value of multimodal MRI, including amide proton transfer-weighted imaging (APT), diffusion kurtosis imaging (DKI), and T2 mapping sequences for estimating human epidermal growth factor receptor-2 (Her-2) expression in patients with endometrial cancer (EC). Methods: A total of 54 patients with EC who underwent multimodal pelvic MRI followed by biopsy were retrospectively selected and divided into the Her-2 positive (n = 24) and Her-2 negative (n = 30) groups. Her-2 expression was confirmed by immunohistochemistry (IHC). Two observers measured APT, mean kurtosis (MK), mean diffusivity (MD), and T2 values for EC lesions. Results: The Her-2 (+) group showed higher APT values and lower MD and T2 values than the Her-2 (−) group (all *p* < 0.05); there was no significant difference in MK values (*p* > 0.05). The area under the receiver operating characteristic curve (AUC) of APT, MD, T2, APT + T2, APT + MD, T2 + MD, and APT + MD + T2 models to identify the two groups of cases were 0.824, 0.695, 0.721, 0.824, 0.858, 0.782, and 0.860, respectively, and the diagnostic efficacy after combined APT + MD + T2 value was significantly higher than those of MD and T2 values individually (*p* = 0.018, 0.028); the diagnostic efficacy of the combination of APT + T2 values was significantly higher than that of T2 values separately (*p* = 0.028). Weak negative correlations were observed between APT and T2 values (r = −0.365, *p* = 0.007), moderate negative correlations between APT and MD values (r = −0.560, *p* < 0.001), and weak positive correlations between MD and T2 values (r = 0.336, *p* = 0.013). The APT values were independent predictors for assessing Her-2 expression in EC patients. Conclusion: The APT, DKI, and T2 mapping sequences can be used to preoperatively assess the Her-2 expression in EC, which can contribute to more precise treatment for clinical preoperative.

## 1. Introduction

Endometrial cancer (EC) is the 4th most common malignancy in women. Its occurrence and mortality rates have risen worldwide, particularly in younger populations [1]. Surgery, which includes removing fallopian tubes, the uterus, and ovaries, is considered the most effective treatment for EC. A combination of radiotherapy, chemotherapy, and hormonal therapy is used for patients with advanced-stage tumors [2]; however, some patients are prone to recurrence after therapy. Recent data have shown that molecular therapy targeting the human epidermal growth factor receptor-2 (Her-2) gene is of significant benefit in the clinical diagnosis and improvement of the prognosis of EC patients [3]. As a tyrosine kinase receptor, the Her-2 gene can stimulate oncogenesis, invasion, and metastasis through gene amplification and protein overexpression [4]. Nevertheless, screening for the Her-2 gene requires a biopsy, which is not always well accepted by patients, considering it is an invasive approach. Also, the limitations to such invasive procedures include difficulty in acquiring tumor samples for both tumor quantity and quality.

With the development of multi-parameter magnetic resonance imaging (mpMRI), quantitative parameters have been applied for assessing uterine disease [5]. The amide proton transfer-weighted (APTw) imaging [6] is a novel contrast media-free molecular MRI technique that can detect the exchange rate of water molecules with amide protons in proteins and peptides. APTw can differentiate type I and II EC, predict type I EC risk factors, and study microsatellite instability in endometrial cancer [7,8,9]. 

Diffusion kurtosis imaging (DKI) is another imaging technique based on the non-Gaussian distributed diffusive motion of water molecules that can quantitatively reflect the complexity of tissue microstructure by several parameters such as mean kurtosis (MK) and mean diffusivity (MD) [10]. DKI sequence is an imaging technique for estimating lesion heterogeneity, which has been used in research to evaluate the extent of endometrial fibrosis and predict lymphatic vascular invasion in cervical cancer [11,12].

T2 mapping imaging reflects tumor microstructural information by detecting the water molecule content of tissue cells [13]. Previous works have demonstrated the use of this sequence in assessing the pathological features of cervical cancer [5]. 

In this study, we assessed the value of multimodal MRI, including APTw, DKI, and T2 mapping sequences for estimating Her-2 gene expression in EC. These data may provide auxiliary information for clinical guidance of EC patient treatment and prognostic assessment.

## 2. Materials and Methods

### 2.1. Participants

This study was approved by the Medical Ethics Committee of our hospital, and informed consent was exempt for the included patients. Our ethics committee approved this research and exempted patients from informed consent. 

**Figure 1:**
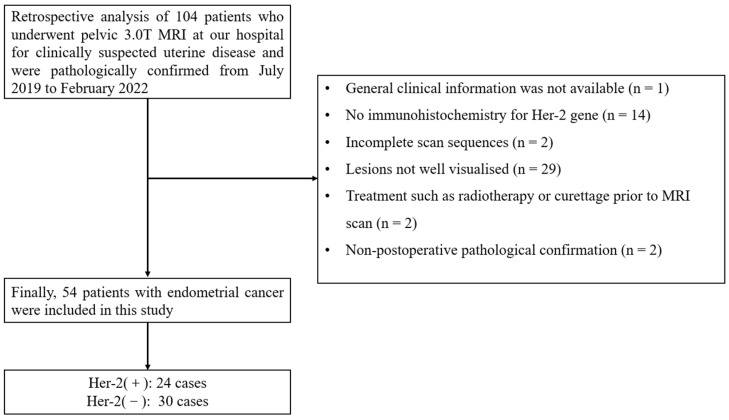
The study flow chart.

A total of 104 patients with clinically suspected uterine lesions who underwent pelvic 3.0T MRI Philips 3.0T MRI (Ingenia CX, Philips Healthcare, Best, The Netherlands) in our hospital between July 2019 and February 2022 were selected. The inclusion criteria were: (1) EC confirmed by postoperative pathology (including Her-2 expression status) within 2 weeks after MRI and postoperative immunohistochemical testing; (2) completely scanned sequences (including APTw, DKI and T2 mapping sequences); (3) good image quality, with no obvious artifacts. Exclusion criteria were: (1) lesion maximum diameter < 1.0 cm; (2) incomplete general clinical information (lesion stage is unclear); (3) radiotherapy or curettage were used before the MRI scan. Finally, patients were divided into the Her-2-positive group (24 cases, aged 36 to 79 years, with a mean age of (58.5 ± 10.1) years) and the Her-2 negative group (30 cases, aged 39 to 75 years, with a mean age of (58.0 ± 8.8) years). The flow chart of the enrollment group is shown in Figure 1.

### 2.2. Clinical Data

Clinical data included age, FIGO stage, menopause, irregular vaginal bleeding (abnormal vaginal bleeding outside the menstrual cycle), degree of pathological differentiation, lymphovascular interstitial infiltration (confirmed by postoperative pathology), carbohydrate antigen 19-9 (CA-19-9), carbohydrate antigen 125 (CA-125), and human epididymis protein 4 (HE4).

### 2.3. MRI Imaging

A 3.0T MRI scanner was used for pelvic examination with a 32-channel abdominal coil. Patients were instructed to empty their bladders and abstain from food for 4–6 h before the scan; intrauterine devices were removed 1d before the examination. The scan sequence included T2WI, APTw, DKI, DWI (b = 0 and 800 s/mm^2^), and T2 mapping; the scan parameters are shown in Table 1. APT images were obtained using a 3D turbo spin-echo sequence, continuous RF pulses with a saturation power of 2.0 μT, and an amide proton saturation time of 2 s. The frequency of water is located at 0 ppm of the Z-spectrum. Data were acquired using seven saturation frequency offsets (±2.7, ±3.5, ±4.3, and −1540 ppm), and the Z-spectrum was fitted to each data point. In addition, B0 plots were obtained from three echo acquisitions at +3.5 ppm for B0 correction. In the final post-processed APT images, the APT value was determined by the magnetization transfer ratio with asymmetric analysis (MTRasym) at +3.5 ppm as [14]:

APT (%) = MTRasym[Δω = +3.5 ppm](%)(1)

DKI adopted an axial 2D echo-planar diffusion-weighted sequence with 3b values (0, 1000, 2000 s/mm^2^) to obtain the quantitative parameters MK and MD values [15]. The DKI parameters are formulated as follows:

S(b) = S0 × exp (−b × MD + b2 × MD2 × MK/6) (2)

where S and S0 are the signal intensity at a certain b value and b value of 0, respectively; the mean diffusion coefficient (MD value) is the corrected apparent diffusion coefficient; the mean kurtosis (MK value) represents the degree of deviation from the Gaussian distribution.

The T2 mapping was acquired with the Gradient Spin Echo (GraSE) technique using 5 spin echoes (TE1 = 20 ms and delta TE = 20 ms). For T2 mapping, signal from each voxel was fitted with the following equation: $S_{i}=M_{0}e^{-{TE}_{i}/T2}$, where S0 and S_i_ are the signal intensities acquired at echo times of 0 and ith TE.

### 2.4. Imaging Analysis

**Figure 2:**
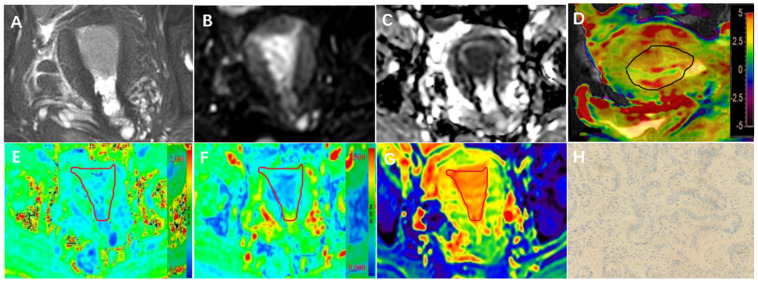
A 73-year-old with EC Her-2 (−) confirmed by immunohistochemistry. (**A**–**H**) T2WI (**A**), DWI (**B**), ADC (**C**), Sagittal T2WI and APT image fusion (**D**), MK (**E**), MD (**F**), and T2 mapping map (**G**) showed endometrial thickening; APT, MK, MD, and T2 values were 2.7%, 0.514, 0.775 μm^2^/ms, and 79.03 ms, respectively. A Her-2 immunohistochemical map of tumor tissue (H) (HE × 200). (**H**) On light microscopy, Her-2 staining was negative.

**Figure 3:**
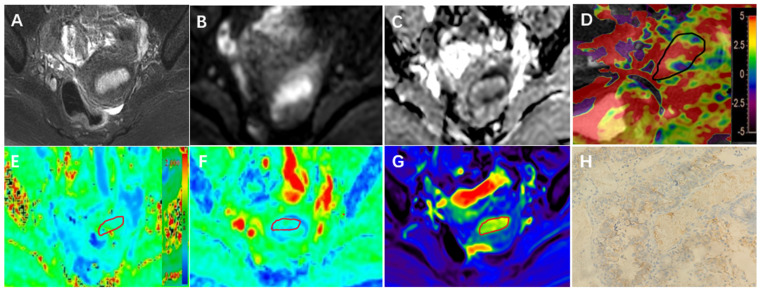
A 66-year-old with EC Her-2 (+) confirmed by immunohistochemistry. (**A**–**H**) T2WI (**A**), DWI (**B**), ADC (**C**), Sagittal T2WI and APT image fusion (**D**), MK (**E**), MD (**F**), and T2 mapping map (**G**) showed endometrial thickening; APT, MK, MD, and T2 values were 2.60%, 0.623, 1.13 μm^2^/ms, and 98.34 ms, respectively. A Her-2 immunohistochemical map of tumor tissue (**H**) (HE × 200). (**H**) On light microscopy, the tumor cell membrane showed brownish-yellow staining.

The raw DWI images, APTw images, DKI images, and T2 mapping images obtained after scanning were transferred to Intellispace Portal v10.0 workstation (Philips Healthcare) for post-processing which generated ADC, APT, MK, MD, and T2 mapping pseudo-color images. All enrolled cases underwent conventional sagittal and axial T2 weighted imaging (T2WI) scans. DWI, DKI, and T2 mapping maintained the same scanning orientation with axial T2WI, and for these sequences, the ROI delineation was performed with reference to axial T2WI. APTw imaging maintained the same scanning orientation with sagittal T2WI, and for APTw, the ROI delineation was performed with reference to sagittal T2WI. Two radiologists (with 7 and 2 years of diagnostic experience, respectively) identified the tumor site based on T2WI, DWI, and ADC images. In case of disagreement between the two doctors, the region of interest (ROI) was outlined at the largest level of the solid lesion, and the ROI was copied to the corresponding levels of the APT, MD, MK, and T2 mapping images to avoid cystic lesions, necrosis, and hemorrhage, as shown in Figure 2 and Figure 3.

### 2.5. Pathological Analysis

The Her-2 expression status of the enrolled cases was independently adjusted by two diagnostic pathologists (with 3 and 5 years of diagnostic experience, respectively) using a double-blind method. Any disagreements were resolved by discussion. The immunohistochemistry (IHC) analysis process was as follows: first, tissues were collected by biopsy, fixed with formalin, dehydrated, and then sectioned from wax blocks (3–5 µm thick sections). Next, the thermal repair was performed after dehydration and hydration. Consequently, samples were treated with 3% H_2_O_2_ solution, incubated for 20 min, and washed three times with phosphate-buffered saline (PBS) for 5 min each. They were then incubated overnight with primary antibody (Her-2, Affinity Biosciences, Dalian, China) at 4 °C, washed three times with PBS, and then treated with biotin-labeled secondary antibody for 30 min at 37 °C. Slides were then restained with hematoxylin, fixed with neutral gum, and observed under a light microscope. IHC results were scored as follows [13]: negative is determined by a Her-2 of 0 or 1+, and positive when Her-2 is 3+. Positive Her-2 gene expression was defined as the presence of brownish-yellow granules or high staining of tumor cell membranes.

### 2.6. Statistical Analysis

Statistical analysis was performed using SPSS 26.0 and Medcala 15. The Interclass correlation coefficient (ICC) was used to assess the concordance between the two observers. The Shapiro–Wilk test was used to estimate the normality of APT values, MD, MK, and T2 values. Data conforming to a normal distribution were expressed as x ± s and non-normally distributed data were expressed as 50% (25%, 75%). An independent samples *t*-test or Mann–Whitney U-test was used to assess the differences between the parameters in the two groups of cases. The discrimination efficacy of the differential parameter values was analyzed using the receiver operating characteristic curve (ROC), and sensitivity, specificity, and threshold values were recorded. The predicted outcomes of the joint model with differential parameters were calculated using binary logistic regression. De-Long tests were adopted to compare the differences between the ROCs of each difference parameter. General clinical data (age, FIGO stage, menopause, clinical symptoms (irregular vaginal bleeding), degree of pathological differentiation, lymphovascular interstitial infiltration, CA-199, CA-125, and HE4 differences between the two groups were analyzed using the independent samples *t*-test, chi-square test, and Fisher’s exact probability method. The association between Her-2 expression status and risk factors was analyzed using univariate and multifactorial logistic regression. A variance inflation factor was used to test the covariance of the quantitative parameter values. The correlation between two separate parameters was measured using the Spearman correlation test.

## 3. Results

### 3.1. General Clinicopathological Data Comparison

Age, FIGO stage, menopause, irregular vaginal bleeding (abnormal vaginal bleeding outside the menstrual cycle), degree of pathological differentiation, lymphovascular interstitial infiltration (postoperative pathology confirmed that cancer cells invaded lymphatic vessels), CA-199, CA-125, and HE4 of the two groups were analyzed. The degree of EC pathological differentiation was significantly different between the two groups (*p* < 0.05), while there was no difference in other clinicopathological data (*p* > 0.05) (Table 2).

### 3.2. The Consistency Analysis of Each Parameter Measured by Two Observers

The interobserver agreement analysis of the APT, MK, MD, and T2 values measured by the two observers showed excellent agreement (ICC > 0.75) for all measured parameters, as shown in Table 3.

### 3.3. Comparison of the Differences in the Parameters between the Two Groups

**Figure 4:**
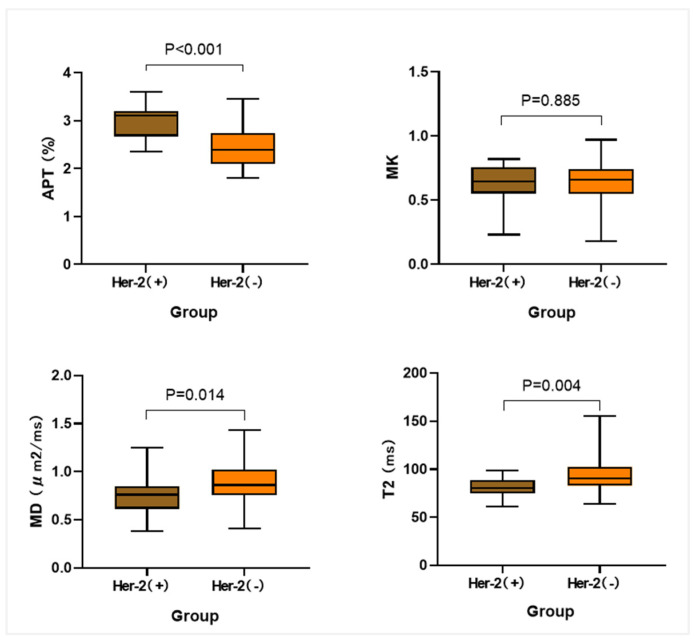
Comparison of parameter values between cases in the Her-2 (+) and Her-2 (−) groups.

The mean values of APT, MD, and MK measured by both observers had normal distribution, and the mean value of T2 had non-normal distribution. The results showed that the Her-2 positive group had higher APT values than the negative group and lower MD and T2 values (all *p* < 0.05), while the differences in MK values between the Her-2 positive and negative groups were not statistically significant (*p* > 0.05), as shown in Table 4 and Figure 4.

### 3.4. The Identification Efficiency of Each Parameter and Comparison

**Figure 5:**
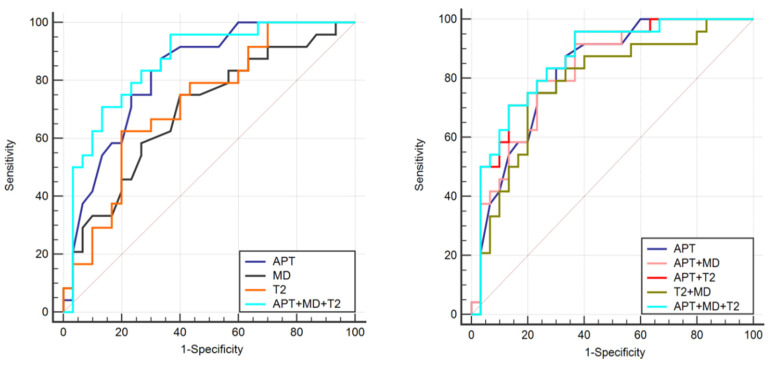
ROC curve of APT, MD, T2, and combined parameter model to identify the two groups of cases.

The AUCs for APT value, MD value, T2 value, APT + T2 value, APT + MD values, T2 + MD values, and the combined APT + MD + T2 values model to identify the Her-2 positive and Her-2 negative was 0.824, 0.695, 0.721, 0.824, 0.858, 0.782, and 0.860, respectively, as shown in Table 5 and Figure 5. The De-Long test showed a statistically significant difference in AUC values between the combined APT + T2 model and T2 values alone (Z = 2.186, *p* = 0.028); the combined APT + MD + T2 model was significantly different from MD and T2 values alone (Z = 2.363, 2.196, *p* = 0.018, 0.028), as shown in Table 6. 

### 3.5. Correlation Analysis of the Parameters 

**Figure 6:**
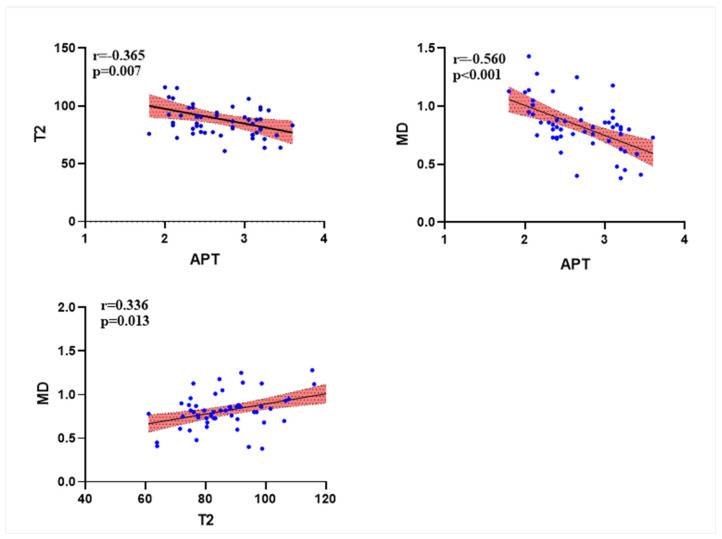
Correlation analysis graph for each individual difference parameter.

The APT values showed a low negative correlation with T2 values (r = −0.365, *p* = 0.007), the APT values showed a moderate negative correlation with MD values (r = −0.560, *p* < 0.001), and the MD values showed a low positive correlation with T2 values (r = 0.336, *p* = 0.013) (Figure 6).

### 3.6. Regression Analysis

The regression analysis was performed on the degree of differentiation and the values of each parameter (APT value, MK value, MD value, and T2 value). The univariate logistic regression showed that the degree of differentiation, APT, MD, and T2 values were associated with Her-2 expression in EC. In addition, multivariate logistic regression showed that APT values were independent predictors for evaluating Her-2 gene expression in EC (Table 7). The results of covariance test showed that the variance inflation factor (VIF) of APT, MK, MD, and T2 values were 1.000, 1.000, 1.426, and 1.426, respectively, which were <5, indicating that the factors were independent of each other.

## 4. Discussion

The biological behavior of tumors, including excessive proliferation and the ability to invade and metastasize tissues, is regulated by multiple factors. It has been shown that amplification of the Her-2 gene often leads to increased expression of the Her-2 protein, which, in turn, activates the phosphatidylinositol-4,5-bisphosphate 3-kinase (PI3K) axis and mitogen-activated protein kinase (MAPK) cascade to induce cancer cell growth and invasion [13]. Moreover, the tumor microenvironment also has an important role in the development and progression of malignant tumors as it can influence the transformation of the tumor phenotype, increasing malignant tumor heterogeneity [14]. Yang et al. [14] found more tumor infiltration lymphocytes (TILs) in Her-2-positive regions of breast cancer tumor cells than in Her-2-negative expression regions. In this study, we found that Her-2 positive patients tended to have lower differentiation and higher tumor grade, which may be related to the high expression of CD4+ T cells as well as the relatively low expression of CD8+ T cells in the tumor stroma of the Her-2 positive group [16]. Furthermore, Yang et al. [17,18] have confirmed that Her-2 positivity is usually indicative of EC with high proliferation and low differentiation.

Immunohistochemistry is considered the gold standard for evaluating Her-2 expression. Nevertheless, to perform a pathological examination, tissue must be obtained by biopsy, which is not always well accepted by the patient and may not provide enough samples for analysis [19,20]. Thus, searching for real-time non-invasive methods for assessing Her-2 expression may be very useful. Li et al. [19] used DCE-MRI imaging histology to predict the Her-2 expression status of breast cancer, but the process is complex, limiting its widespread use in clinical practice. Park et al. [20] suggested using F2-FDG (18F) [2-fluoro-2-deoxyglucose] positron emission tomography (PET)/computed tomography (CT) for Her-2 gene screening for assessing the prognosis of patients with stage VI gastric cancer treated with trastuzumab. However, PET-CT examinations are expensive, with a high radiation dose and possible false positive results.

Quantitative parametric imaging in MRI allows preoperative non-invasive assessment of the expression of EC Her-2 gene status. As a non-invasive MR imaging technique at the molecular level, APTw can reflect the metabolic and pathophysiological information of the lesion by measuring the quantitative parameter APT value [21,22]. Meanwhile, APTw signals can reflect protein and peptide content of tumor tissue [23], which increases with increased cell density, heterotypy, microvascular density (MVD), and microscopic necrosis of tumor tissue [24,25], resulting in increased APT values. In addition, the increase in PH of the tumor microenvironment caused by hypoxia also contributes to the increase in APT values [26]. In previous studies, Tian et al. [7] have demonstrated the value of APTw in differentiating stage IA endometrial cancer from endometrial polyps with a diagnostic efficacy of 0.850. In addition, several studies have shown [27,28,29,30] that APTw is valuable for differentiating benign and malignant endometrial disease, assessing Ki-67 expression, preoperative risk assessment, and evaluating microsatellite instability status.

In this study, the APT values in the Her-2 positive group were higher than those in the Her-2 negative group, presumably for the following three reasons: first, positive expression of the Her-2 gene can cause abnormal proliferation of tumor cells, which, in turn, causes malignant invasion of tumor cells [31]. Compared to the Her-2 negative group, the Her-2 positive group was less differentiated and had increased tumor cell density, microscopic necrosis, and PH changes, as well as more active tumor tissue metabolism and higher protein and peptide content, which led to faster rates of amide proton transfer and higher APT values [24]. In addition, the increased degree of nuclear heterotypy in the Her-2 positive expression group can facilitate the interaction of macromolecules with the cell membrane, which in turn promotes the release of proteins and peptides [9]. Eventually, the Her-2 gene increases the invasiveness of the tumor by upregulating the permeability of the vascular endothelium and tumor vessels [31], while the increase in neovascularization and the increase in blood perfusion causes an increase in plasma protein and hemoglobin content, ultimately leading to an increase in APT values [32]. Tian et al. [22] evaluated EC Her-2 gene expression levels using APTw and mDixon-Quant sequences, showing that the diagnostic efficacy of the APT and R2* values were 0.755 and 0.739, respectively, which is consistent with the results of the present study, and further validates the feasibility of APT imaging for assessing EC Her-2 gene expression.

In contrast to the traditional concept of free diffusion of water in an unconstrained environment, DKI describes the diffusion process in a more objective microenvironment by analyzing the movement of water molecules in different directions, which makes it more feasible to probe the non-Gaussian diffusion properties of complex components and structural organization [33]. It has been shown that the diagnostic accuracy of quantitative DKI parameters is superior to DWI imaging [34]. In addition, Jin et al. [8] used this sequence to demonstrate that MD and MK values are independent risk factors for the one stage of EC.

The present study explored MK and MD values to assess Her-2 expression in EC patients. MK is the mean value of kurtosis in all diffusion directions, which mainly reflects the extent to which the movement of water molecules deviates from the Gaussian distribution and is a parameter for assessing tumor cell heterogeneity [35]. Our data showed no difference between the MK values of the Her-2 positive and negative groups, which may be because both groups have malignant characteristics, and the tumor microenvironment is altered due to abnormal proliferation of tumor cells and micro angiogenesis in both groups, which ultimately causes the water molecule dispersion to deviate from the Gaussian distribution in both groups. However, we found lower MD values in the Her-2 positive group compared to Her-2 negative group. The MD value is the corrected diffusion coefficient of water molecules [36], which can objectively reflect the diffusion restriction of tissue cells. Since Her-2 positivity promotes EC cell proliferation and inhibits apoptosis [37], the tumor cells in the Her-2 positive group are relatively denser and have more significant water molecule diffusion restriction; meanwhile, the increased lack of oxygen in the tumor microenvironment decreases the PH value. Ultimately, a decrease in the pH of the tumor microenvironment may lead to an increase in APT values. We also found a moderate negative correlation between APT and MD values.

T2 mapping sequences can also reflect information about water molecules in the microenvironment of tumor cells through T2 values [38], which is useful for differentiating tubular from non-tubular adenocarcinoma of the rectum, with a diagnostic sensitivity and specificity of 100% and 97%, respectively [38]. In addition, Ma et al. [39] found that T2 mapping sequences can predict microsatellite instability status in EC patients with a diagnostic efficacy of 0.780. The present study used T2 mapping to assess its value in distinguishing between the two groups of cases. It was found that the T2 values of EC in the Her-2 expression positive group were lower than those in the negative group, which is in line with Fujima’s study [40]. These findings could be due to EC in the Her-2-positive group, which had a greater capacity for cell proliferation and lymphovascular infiltration, so the increased density of tumor cells reduced the intercellular water content [41]. At the same time, an increase in tumor cell density may lead to a decrease in MD values (caused by restricted diffusion of water molecules) and an increase in APT values (caused by increased protein and peptide content and decreased PH). Our results also confirmed a low positive correlation between T2 values and MD values, whereas T2 values had a low negative correlation with APT values. In our previous discussion, we suggested that the Her-2 positive expression group had increased MVD and microscopic tumor necrosis [24] and that the above factors might increase the water content between tumor cells, increasing T2 values. However, our experimental results ultimately showed that the T2 values of EC cells in the Her-2 positive group were smaller than those in the Her-2 negative group, indicating that in the Her-2 positive group, tumor cells proliferated more than necrosis.

In the present study, the diagnostic efficacy of APT + T2 was significantly higher than that of T2 alone, and the diagnostic efficacy of the combination of APT + MD + T2 was significantly higher than that of MD and T2 alone, reflecting the importance of APT in the combined model for disease diagnosis. Moreover, APT was an independent predictor of EC Her-2 expression, further reflecting the unique value of APTw in assessing lesions from a molecular imaging perspective. Finally, the diagnostic efficacy of the combined model of APT + MD + T2 values was 0.860, which indicates that the APT, DKI, and T2 mapping sequences complement each other, and that the combination of the three has some information enrichment value. Moreover, the purpose of this study is to explore the value of multi-parametric imaging in preoperative assessment of Her-2 gene expression in EC patients. Of course, immunohistochemical indicators that reflect vascular microperfusion and hypoxia, such as MVD and VEGF, can also affect the prognosis and tumors of EC patients, but they were not within the scope of current study. And it will be of great interest for future studies to include the extra immunohistochemical indicators.

There are some limitations in the present study: (1) the layers of APT, DKI, and T2 mapping images did not fully correspond to each other, which may cause some bias in the measured parameters; (2) this study only outlined the largest level of the tumor entity, but not the whole tumor domain, so it is difficult to assess the heterogeneity of the tumor. Future histological studies are proposed to assess the value of the two sequences on the expression level of EC Her-2. (3) The samples volume of this study is small and further collection is to be followed up. (4) There was no serous endometrial cancer in the cases included in this study, and only one case of carcinosarcoma and poorly differentiated carcinoma was included, which will be investigated in a subsequent study after enlarging the sample size to assess the value of multimodal imaging in serous endometrial cancer. (5) Voxel correction of 3DAPT images was not performed in this study.

In summary, the APTw, DKI, and T2 mapping sequences can be used for non-invasive evaluation of EC Her-2 gene expression at the tumor cell microenvironment and molecular metabolism level, which has a certain clinical application value.

## Figures and Tables

**Table 1:** MRI scan sequences and parameters.

Parameters	Scan Time (min s)	TR (ms)	TE (ms)	Matrix	FOV (mm^2^)	Thickness (mm)	Gap (mm)
T2WI	1 min 27 s	3363	87	312 × 312	250 × 250	4.0	1.0
DWI	1 min 11 s	2615	61	60 × 70	220 × 220	4.0	1.0
3D APTw	4 min 5 s	5174	8	64 × 45	130 × 130	5.0	1.0
DKI	5 min 19 s	1904	84	129 × 118	380 × 356	5.0	1.0
T2 mapping	17 s	1157	100	192 × 115	70 × 319	7.0	2.0

DKI with b value of 0, 1000, 2000 s/mm^2^. T2WI = T2-weighted imaging; TR = time of repetition; TE = echo time; FOV = field of view; TRA = transection; DWI = diffusion-weighted imaging; APTw = amide proton transfer-weighted; DKI = diffusion kurtosis imaging. For the conventional DWI sequence, the NSA for acquisitions with b values of 0 and 800 s/mm^2^ were 1 and 1, respectively; for the DKI sequence, the NSA used for acquisitions with b values of 0, 1000, and 2000 s/mm^2^ were 1, 1, and 2, respectively.

**Table 2:** The clinicopathological information.

Clinicopathological Features	Her-2 (+) (n = 24)	Her-2 (−) (n = 30)	*p*
Age (year)	58.7 ± 10.3	58.3 ± 8.9	0.897 ^a^
Size (cm)			
FIGO stage			0.891 ^b^
I	18	22	
II	2	3	
III	3	5	
IV	1	0	
Menopausal conditions			0.483 ^b^
Before	3	7	
After	21	23	
Irregular vaginal bleeding			1.000 ^b^
Yes	22	27	
No	2	3	
Differentiation degree			0.003 ^b^
High	2	13	
Medium	11	13	
Low	11	4	
Lymphovascular interstitial infiltration			0.839 ^c^
Yes	7	8	
No	17	22	
CA-199			0.267 ^c^
Positive Negative	8 16	6 24	
CA-125			0.546 ^c^
Positive Negative	4 20	7 23	
HE4			0.851 ^c^
Positive Negative	15 9	18 12	

^a^ represents data analyzed using the independent samples *t*-test; ^b^ represents data analyzed using Fisher’s exact probability method; ^c^ represents data analyzed using the chi-square test. CA-19-9 = cabobydrale anigen 19-9; CA-125 = carbohydrate antigen 125; HE4 = human epididymis protein 4.

**Table 3:** Agreement analysis of the values of each parameter measured by the two observers.

	Observer 1	Observer 2	ICC
Her-2 (+) patients (n = 24)			
APT (%)	2.92 ± 0.37	3.01 ± 0.37	0.798
MK	0.63 ± 0.14	0.64 ± 0.15	0.964
MD (μm^2^/ms)	0.734 ± 0.21	0.75 ± 0.20	0.980
T2 (ms)	81.21 ± 9.48	81.20 (76.09, 89.50)	0.812
Her-2 (−) patients (n = 30)			
APT (%)	2.36 ± 0.50	2.46 ± 0.49	0.896
MK	0.63 ± 0.17	0.61 ± 0.16	0.976
MD (μm^2^/ms)	0.88 ± 0.21	0.89 ± 0.20	0.957
T2 (ms)	91.11 (82.54, 99.95)	90.26 (82.78, 104.18)	0.987

APT = amide proton transfer; MK = mean kurtosis; MD = mean diffusivity; ICC = Intraclass correlation coefficient.

**Table 4:** Analysis of the differences in the values of each parameter between the two groups of cases.

Parameters	Her-2 (+) Patients (n = 24)	Her-2 (−) Patients (n = 30)	*t*/z	*p*
APT (%)	2.99 ± 0.32	2.41 ± 0.46	4.999	<0.001
MK	0.63 ± 0.14	0.64 ± 0.16	−0.195	0.846
MD (μm^2^/ms)	0.72 ± 0.19	0.86 ± 0.12	−2.771	0.008
T2 (ms)	80.33 (75.00, 88.49)	90.58 (82.71, 102.78)	−2.768	0.006

APT = amide proton transfer; MK = mean kurtosis; MD = mean diffusivity.

**Table 5:** Assessment of the efficacy of each parameter alone or in combination to identify the two groups of cases.

Parameters	AUC (95%CI)	Sensitivity (%)	Specificity (%)	Cutoff
APT	0.824 (0.696, 0.914)	87.5	66.7	2.45%
MD	0.695 (0.555, 0.813)	75.0	60.0	0.82 μm^2^/ms
T2	0.721 (0.582, 0.834)	62.6	80.0	81.81 ms
APT + MD	0.824 (0.696, 0.914)	79.2	76.7	-
APT + T2	0.858 (0.737, 0.938)	95.8	63.3	-
MD + T2	0.782 (0.649, 0.883)	75.0	80.0	-
APT + MD + T2	0.860 (0.738, 0.939)	95.8	63.3	-

APT = amide proton transfer; MK = mean kurtosis; MD = mean diffusivity.

**Table 6:** Results of ROC comparisons for different parameters.

	APT	MD	T2	APT + MD	APT + T2	MD + T2	APT + MD + T2
APT		0.0773	0.2016	1.0000	0.1402	0.5612	0.1332
MD			0.7691	0.0543	0.0219	0.1274	0.0181
T2				0.2030	0.0288	0.1478	0.0281
APT + MD					0.1601	0.5453	0.1375
APT + T2						0.1801	0.7276
MD + T2							0.1637

APT = amide proton transfer; MK = mean kurtosis; MD = mean diffusivity.

**Table 7:** Univariate and multifactorial analysis of EC in HER-2 positive and negative expression groups.

Parameters	Univariate Analyses		Multivariate Analyses	
	OR (95% CI)	*p* Value	OR (95% CI)	*p* Value
Differentiation degree	0.285 (0.113, 0.724)	0.008	0.412 (0.122 1.385)	0.152
APT (%)	1.352 (1.147, 1.592)	<0.001	1.273 (1.056, 1.534)	0.011
MK	0.876 (0.968, 1.038)	0.876	/	/
MD (10^−3^ mm^2^/s)	0.965 (0.936, 0.995)	0.023	1.004 (0.964, 1.045)	0.865
T2 (ms)	0.934 (0.885, 0.985)	0.012	0.941 (0.880, 1.007)	0.077

All factors with *p* < 0.05 in univariate analyses were included in multivariate regression analyses. The bold typeface in the table indicates the logistic regression analyses with statistical significance. APT = amide proton transfer; MK = mean kurtosis; MD = mean diffusivity; OR = odds ratio; CI = confidence interval.

## Data Availability

The datasets generated or analyzed during the study are available from the corresponding author upon reasonable request.
